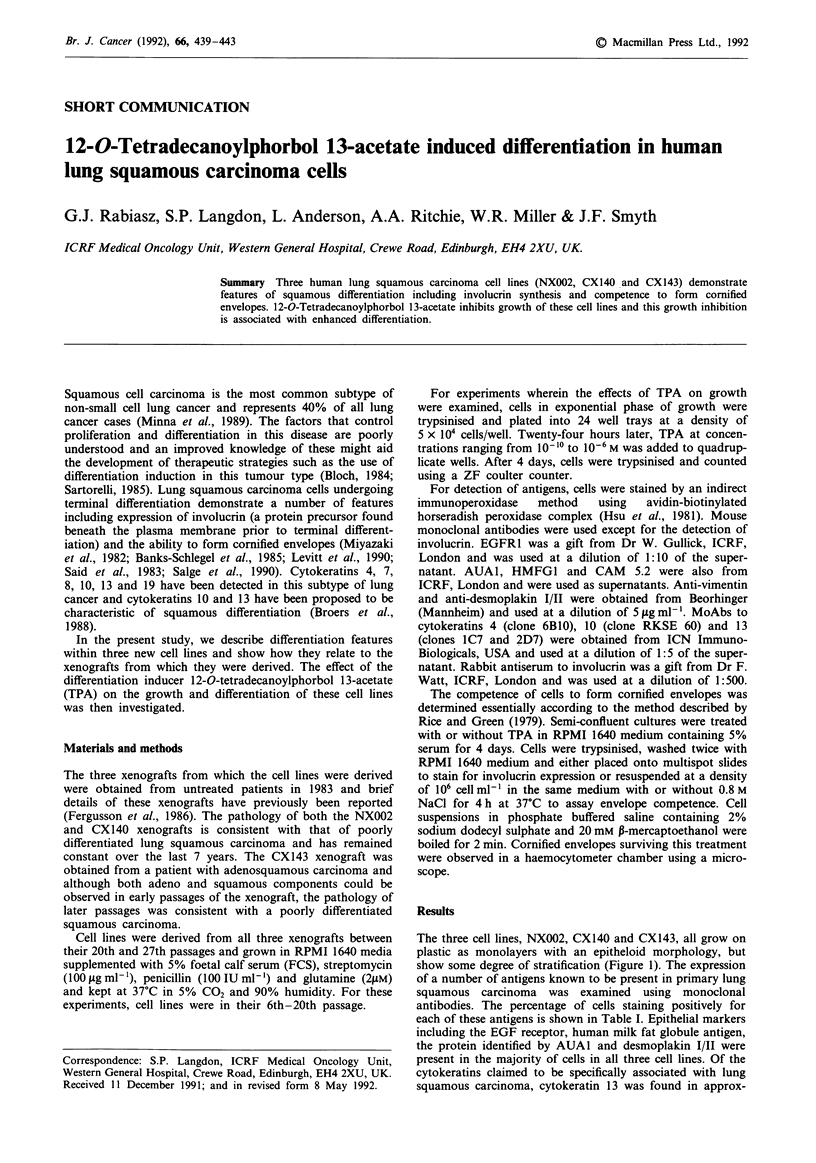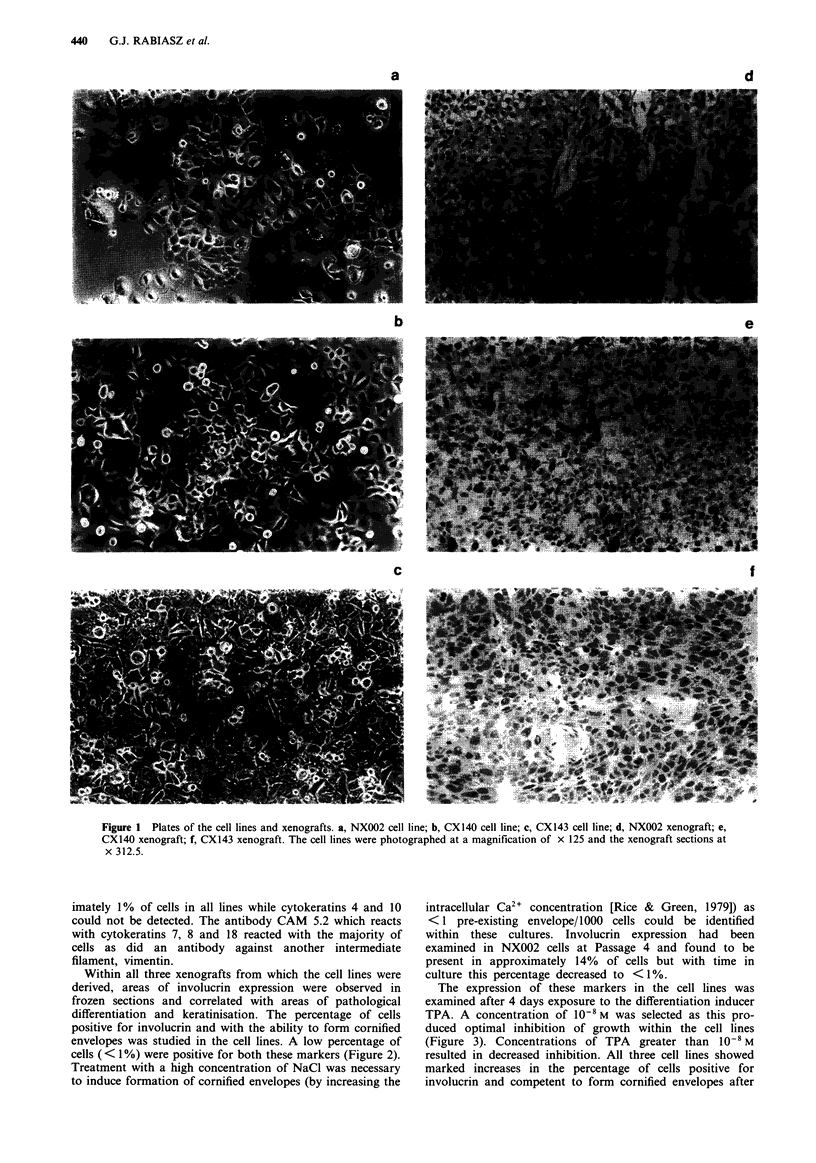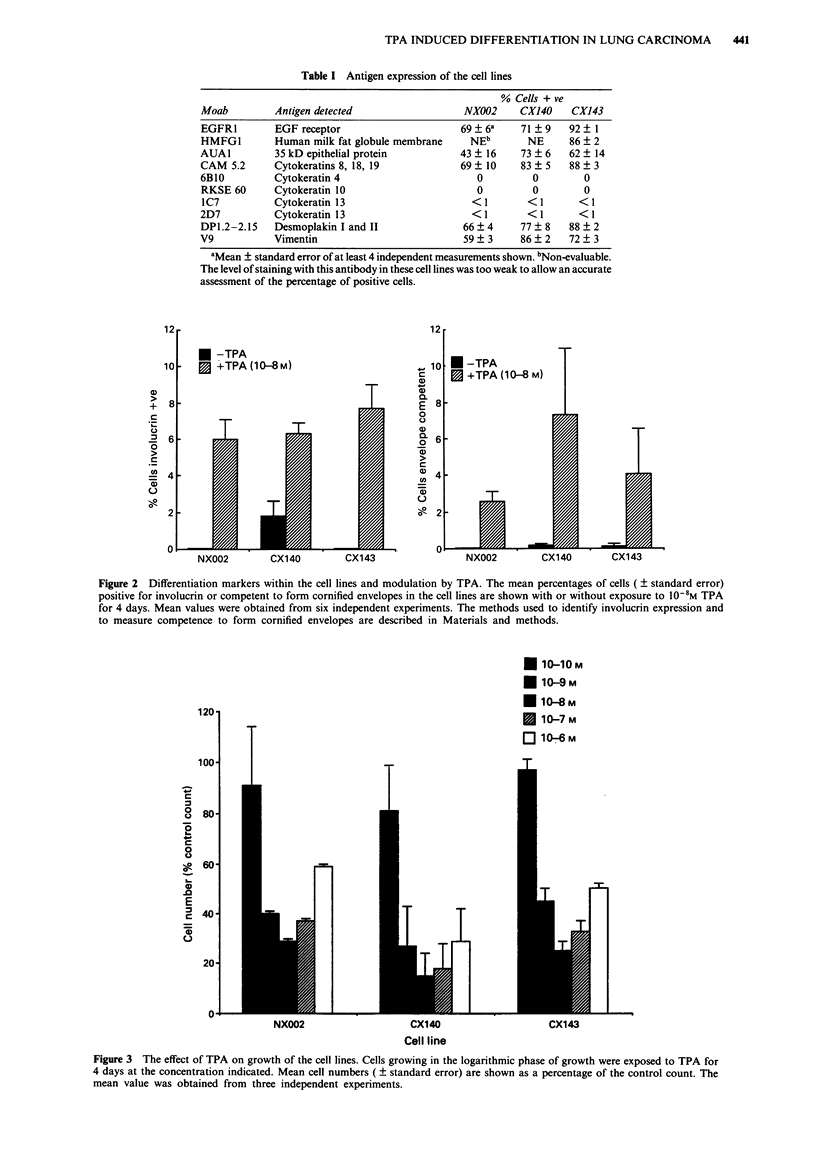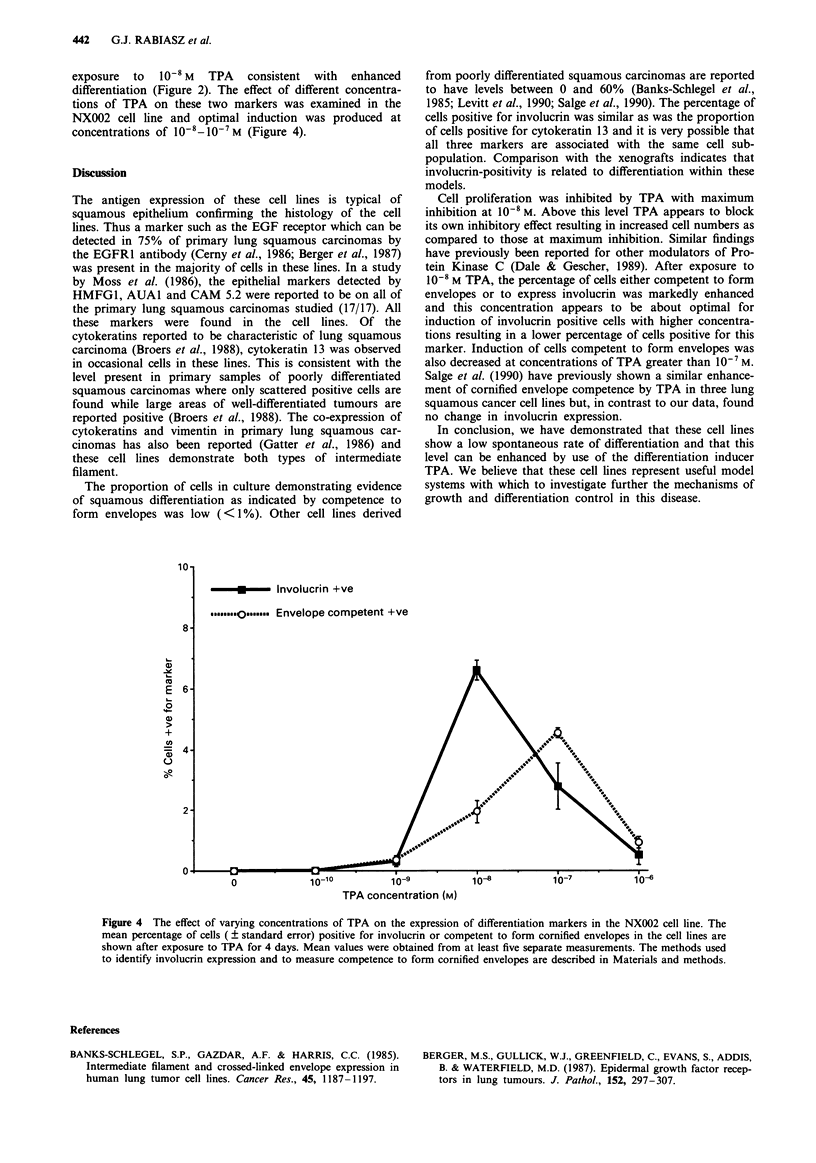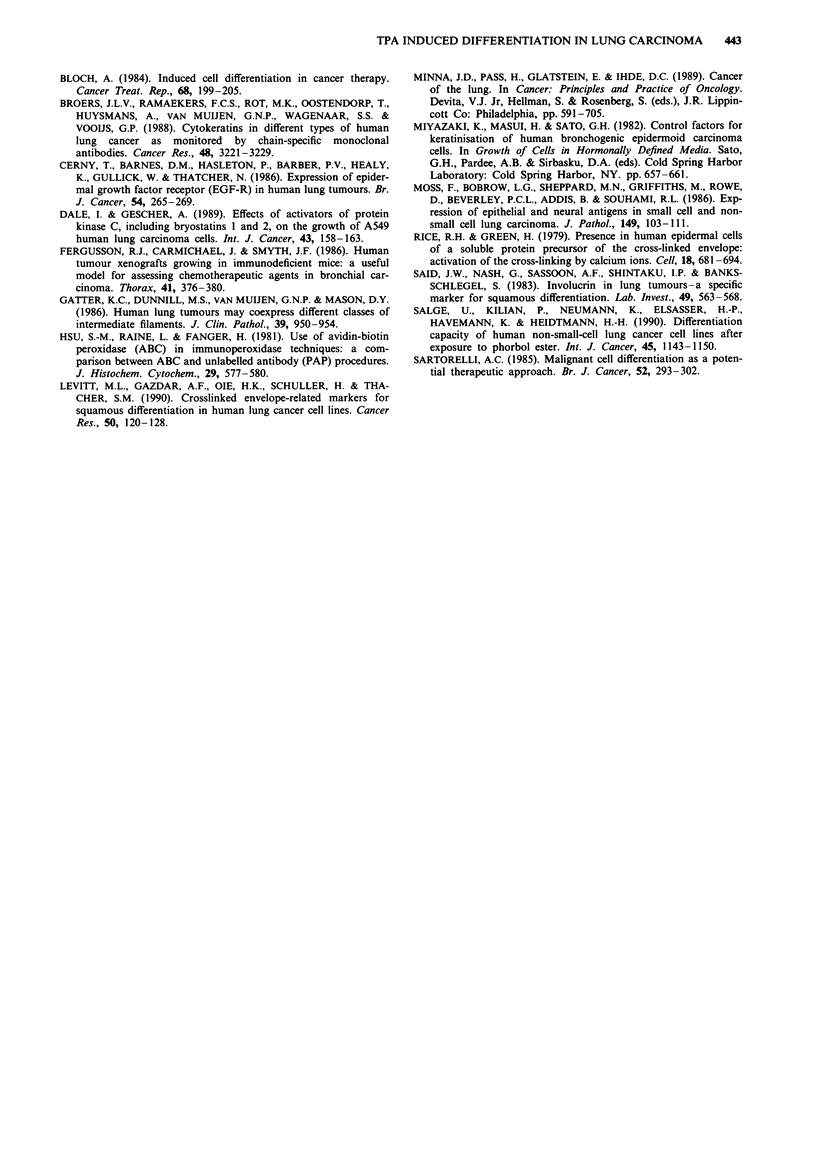# 12-O-tetradecanoylphorbol 13-acetate induced differentiation in human lung squamous carcinoma cells.

**DOI:** 10.1038/bjc.1992.293

**Published:** 1992-09

**Authors:** G. J. Rabiasz, S. P. Langdon, L. Anderson, A. A. Ritchie, W. R. Miller, J. F. Smyth

**Affiliations:** ICRF Medical Oncology Unit, Western General Hospital, Edinburgh, UK.

## Abstract

**Images:**


Br. J. Cancer (1992), 66, 439-443 © Macmillan Press Ltd., 1992

SHORT COMMUNICATION

### 12-*O*-Tetradecanoylphorbol 13-acetate induced differentiation in human lung squamous carcinoma cells

G.J. Rabiasz, S.P. Langdon, L. Anderson, A.A. Ritchie, W.R. Miller & J.F. Smyth

*ICRF Medical Oncology Unit, Western General Hospital, Crewe Road, Edinburgh, EH4 2XU, UK.*

**Summary** Three human lung squamous carcinoma cell lines (NX002, CX140 and CX143) demonstrate features of squamous differentiation including involucrin synthesis and competence to form cornified envelopes. 12-*O*-Tetradecanoylphorbol 13-acetate inhibits growth of these cell lines and this growth inhibition is associated with enhanced differentiation.

Squamous cell carcinoma is the most common subtype of non-small cell lung cancer and represents 40% of all lung cancer cases (Minna *et al.*, 1989). The factors that control proliferation and differentiation in this disease are poorly understood and an improved knowledge of these might aid the development of therapeutic strategies such as the use of differentiation induction in this tumour type (Bloch, 1984; Sartorelli, 1985). Lung squamous carcinoma cells undergoing terminal differentiation demonstrate a number of features including expression of involucrin (a protein precursor found beneath the plasma membrane prior to terminal differentiation) and the ability to form cornified envelopes (Miyazaki *et al.*, 1982; Banks-Schlegel *et al.*, 1985; Levitt *et al.*, 1990; Said *et al.*, 1983; Salge *et al.*, 1990). Cytokeratins 4, 7, 8, 10, 13 and 19 have been detected in this subtype of lung cancer and cytokeratins 10 and 13 have been proposed to be characteristic of squamous differentiation (Broers *et al.*, 1988).

In the present study, we describe differentiation features within three new cell lines and show how they relate to the xenografts from which they were derived. The effect of the differentiation inducer 12-*O*-tetradecanoylphorbol 13-acetate (TPA) on the growth and differentiation of these cell lines was then investigated.

### Materials and methods

The three xenografts from which the cell lines were derived were obtained from untreated patients in 1983 and brief details of these xenografts have previously been reported (Fergusson *et al.*, 1986). The pathology of both the NX002 and CX140 xenografts is consistent with that of poorly differentiated lung squamous carcinoma and has remained constant over the last 7 years. The CX143 xenograft was obtained from a patient with adenosquamous carcinoma and although both adeno and squamous components could be observed in early passages of the xenograft, the pathology of later passages was consistent with a poorly differentiated squamous carcinoma.

Cell lines were derived from all three xenografts between their 20th and 27th passages and grown in RPMI 1640 media supplemented with 5% foetal calf serum (FCS), streptomycin (100 $\mu$g ml$^{-1}$), penicillin (100 IU ml$^{-1}$) and glutamine (2$\mu$M) and kept at 37°C in 5% $CO_2$ and 90% humidity. For these experiments, cell lines were in their 6th–20th passage.

For experiments wherein the effects of TPA on growth were examined, cells in exponential phase of growth were trypsinised and plated into 24 well trays at a density of $5 \times 10^4$ cells/well. Twenty-four hours later, TPA at concentrations ranging from $10^{-10}$ to $10^{-6}$ M was added to quadruplicate wells. After 4 days, cells were trypsinised and counted using a ZF coulter counter.

For detection of antigens, cells were stained by an indirect immunoperoxidase method using avidin-biotinylated horseradish peroxidase complex (Hsu *et al.*, 1981). Mouse monoclonal antibodies were used except for the detection of involucrin. EGFR1 was a gift from Dr W. Gullick, ICRF, London and was used at a dilution of 1:10 of the supernatant. AUA1, HMFG1 and CAM 5.2 were also from ICRF, London and were used as supernatants. Anti-vimentin and anti-desmoplakin I/II were obtained from Beorhinger (Mannheim) and used at a dilution of 5 $\mu$g ml$^{-1}$. MoAbs to cytokeratins 4 (clone 6B10), 10 (clone RKSE 60) and 13 (clones 1C7 and 2D7) were obtained from ICN Immuno-Biologicals, USA and used at a dilution of 1:5 of the supernatant. Rabbit antiserum to involucrin was a gift from Dr F. Watt, ICRF, London and was used at a dilution of 1:500.

The competence of cells to form cornified envelopes was determined essentially according to the method described by Rice and Green (1979). Semi-confluent cultures were treated with or without TPA in RPMI 1640 medium containing 5% serum for 4 days. Cells were trypsinised, washed twice with RPMI 1640 medium and either placed onto multispot slides to stain for involucrin expression or resuspended at a density of $10^6$ cell ml$^{-1}$ in the same medium with or without 0.8 M NaCl for 4 h at 37°C to assay envelope competence. Cell suspensions in phosphate buffered saline containing 2% sodium dodecyl sulphate and 20 mM $\beta$-mercaptoethanol were boiled for 2 min. Cornified envelopes surviving this treatment were observed in a haemocytometer chamber using a microscope.

### Results

The three cell lines, NX002, CX140 and CX143, all grow on plastic as monolayers with an epitheloid morphology, but show some degree of stratification (Figure 1). The expression of a number of antigens known to be present in primary lung squamous carcinoma was examined using monoclonal antibodies. The percentage of cells staining positively for each of these antigens is shown in Table I. Epithelial markers including the EGF receptor, human milk fat globule antigen, the protein identified by AUA1 and desmoplakin I/II were present in the majority of cells in all three cell lines. Of the cytokeratins claimed to be specifically associated with lung squamous carcinoma, cytokeratin 13 was found in approx-

Correspondence: S.P. Langdon, ICRF Medical Oncology Unit, Western General Hospital, Crewe Road, Edinburgh, EH4 2XU, UK.
Received 11 December 1991; and in revised form 8 May 1992.

**Figure 1** Plates of the cell lines and xenografts. **a**, NX002 cell line; **b**, CX140 cell line; **c**, CX143 cell line; **d**, NX002 xenograft; **e**, CX140 xenograft; **f**, CX143 xenograft. The cell lines were photographed at a magnification of × 125 and the xenograft sections at × 312.5.

imately 1% of cells in all lines while cytokeratins 4 and 10 could not be detected. The antibody CAM 5.2 which reacts with cytokeratins 7, 8 and 18 reacted with the majority of cells as did an antibody against another intermediate filament, vimentin.

Within all three xenografts from which the cell lines were derived, areas of involucrin expression were observed in frozen sections and correlated with areas of pathological differentiation and keratinisation. The percentage of cells positive for involucrin and with the ability to form cornified envelopes was studied in the cell lines. A low percentage of cells (<1%) were positive for both these markers (Figure 2). Treatment with a high concentration of NaCl was necessary to induce formation of cornified envelopes (by increasing the intracellular $Ca^{2+}$ concentration [Rice & Green, 1979]) as <1 pre-existing envelope/1000 cells could be identified within these cultures. Involucrin expression had been examined in NX002 cells at Passage 4 and found to be present in approximately 14% of cells but with time in culture this percentage decreased to <1%.

The expression of these markers in the cell lines was examined after 4 days exposure to the differentiation inducer TPA. A concentration of $10^{-8}$ M was selected as this produced optimal inhibition of growth within the cell lines (Figure 3). Concentrations of TPA greater than $10^{-8}$ M resulted in decreased inhibition. All three cell lines showed marked increases in the percentage of cells positive for involucrin and competent to form cornified envelopes after

**Table I** Antigen expression of the cell lines

| *Moab* | *Antigen detected* | *% Cells + ve* *NX002* | *CX140* | *CX143* |
|---|---|---|---|---|
| EGFR1 | EGF receptor | 69 ± 6[a] | 71 ± 9 | 92 ± 1 |
| HMFG1 | Human milk fat globule membrane | NE[b] | NE | 86 ± 2 |
| AUA1 | 35 kD epithelial protein | 43 ± 16 | 73 ± 6 | 62 ± 14 |
| CAM 5.2 | Cytokeratins 8, 18, 19 | 69 ± 10 | 83 ± 5 | 88 ± 3 |
| 6B10 | Cytokeratin 4 | 0 | 0 | 0 |
| RKSE 60 | Cytokeratin 10 | 0 | 0 | 0 |
| 1C7 | Cytokeratin 13 | <1 | <1 | <1 |
| 2D7 | Cytokeratin 13 | <1 | <1 | <1 |
| DP1.2–2.15 | Desmoplakin I and II | 66 ± 4 | 77 ± 8 | 88 ± 2 |
| V9 | Vimentin | 59 ± 3 | 86 ± 2 | 72 ± 3 |

[a]Mean ± standard error of at least 4 independent measurements shown. [b]Non-evaluable. The level of staining with this antibody in these cell lines was too weak to allow an accurate assessment of the percentage of positive cells.

**Figure 2** Differentiation markers within the cell lines and modulation by TPA. The mean percentages of cells ( ± standard error) positive for involucrin or competent to form cornified envelopes in the cell lines are shown with or without exposure to $10^{-8}$M TPA for 4 days. Mean values were obtained from six independent experiments. The methods used to identify involucrin expression and to measure competence to form cornified envelopes are described in Materials and methods.

**Figure 3** The effect of TPA on growth of the cell lines. Cells growing in the logarithmic phase of growth were exposed to TPA for 4 days at the concentration indicated. Mean cell numbers ( ± standard error) are shown as a percentage of the control count. The mean value was obtained from three independent experiments.

exposure to $10^{-8}$ M TPA consistent with enhanced differentiation (Figure 2). The effect of different concentrations of TPA on these two markers was examined in the NX002 cell line and optimal induction was produced at concentrations of $10^{-8}-10^{-7}$ M (Figure 4).

### Discussion

The antigen expression of these cell lines is typical of squamous epithelium confirming the histology of the cell lines. Thus a marker such as the EGF receptor which can be detected in 75% of primary lung squamous carcinomas by the EGFR1 antibody (Cerny *et al.*, 1986; Berger *et al.*, 1987) was present in the majority of cells in these lines. In a study by Moss *et al.* (1986), the epithelial markers detected by HMFG1, AUA1 and CAM 5.2 were reported to be on all of the primary lung squamous carcinomas studied (17/17). All these markers were found in the cell lines. Of the cytokeratins reported to be characteristic of lung squamous carcinoma (Broers *et al.*, 1988), cytokeratin 13 was observed in occasional cells in these lines. This is consistent with the level present in primary samples of poorly differentiated squamous carcinomas where only scattered positive cells are found while large areas of well-differentiated tumours are reported positive (Broers *et al.*, 1988). The co-expression of cytokeratins and vimentin in primary lung squamous carcinomas has also been reported (Gatter *et al.*, 1986) and these cell lines demonstrate both types of intermediate filament.

The proportion of cells in culture demonstrating evidence of squamous differentiation as indicated by competence to form envelopes was low (<1%). Other cell lines derived from poorly differentiated squamous carcinomas are reported to have levels between 0 and 60% (Banks-Schlegel *et al.*, 1985; Levitt *et al.*, 1990; Salge *et al.*, 1990). The percentage of cells positive for involucrin was similar as was the proportion of cells positive for cytokeratin 13 and it is very possible that all three markers are associated with the same cell subpopulation. Comparison with the xenografts indicates that involucrin-positivity is related to differentiation within these models.

Cell proliferation was inhibited by TPA with maximum inhibition at $10^{-8}$ M. Above this level TPA appears to block its own inhibitory effect resulting in increased cell numbers as compared to those at maximum inhibition. Similar findings have previously been reported for other modulators of Protein Kinase C (Dale & Gescher, 1989). After exposure to $10^{-8}$ M TPA, the percentage of cells either competent to form envelopes or to express involucrin was markedly enhanced and this concentration appears to be about optimal for induction of involucrin positive cells with higher concentrations resulting in a lower percentage of cells positive for this marker. Induction of cells competent to form envelopes was also decreased at concentrations of TPA greater than $10^{-7}$ M. Salge *et al.* (1990) have previously shown a similar enhancement of cornified envelope competence by TPA in three lung squamous cancer cell lines but, in contrast to our data, found no change in involucrin expression.

In conclusion, we have demonstrated that these cell lines show a low spontaneous rate of differentiation and that this level can be enhanced by use of the differentiation inducer TPA. We believe that these cell lines represent useful model systems with which to investigate further the mechanisms of growth and differentiation control in this disease.

**Figure 4** The effect of varying concentrations of TPA on the expression of differentiation markers in the NX002 cell line. The mean percentage of cells (± standard error) positive for involucrin or competent to form cornified envelopes in the cell lines are shown after exposure to TPA for 4 days. Mean values were obtained from at least five separate measurements. The methods used to identify involucrin expression and to measure competence to form cornified envelopes are described in Materials and methods.

### References

BANKS-SCHLEGEL, S.P., GAZDAR, A.F. & HARRIS, C.C. (1985). Intermediate filament and crossed-linked envelope expression in human lung tumor cell lines. *Cancer Res.*, **45**, 1187–1197.

BERGER, M.S., GULLICK, W.J., GREENFIELD, C., EVANS, S., ADDIS, B. & WATERFIELD, M.D. (1987). Epidermal growth factor receptors in lung tumours. *J. Pathol.*, **152**, 297–307.

BLOCH, A. (1984). Induced cell differentiation in cancer therapy. *Cancer Treat. Rep.*, **68**, 199–205.

BROERS, J.L.V., RAMAEKERS, F.C.S., ROT, M.K., OOSTENDORP, T., HUYSMANS, A., VAN MUIJEN, G.N.P., WAGENAAR, S.S. & VOOIJS, G.P. (1988). Cytokeratins in different types of human lung cancer as monitored by chain-specific monoclonal antibodies. *Cancer Res.*, **48**, 3221–3229.

CERNY, T., BARNES, D.M., HASLETON, P., BARBER, P.V., HEALY, K., GULLICK, W. & THATCHER, N. (1986). Expression of epidermal growth factor receptor (EGF-R) in human lung tumours. *Br. J. Cancer*, **54**, 265–269.

DALE, I. & GESCHER, A. (1989). Effects of activators of protein kinase C, including bryostatins 1 and 2, on the growth of A549 human lung carcinoma cells. *Int. J. Cancer*, **43**, 158–163.

FERGUSSON, R.J., CARMICHAEL, J. & SMYTH, J.F. (1986). Human tumour xenografts growing in immunodeficient mice: a useful model for assessing chemotherapeutic agents in bronchial carcinoma. *Thorax*, **41**, 376–380.

GATTER, K.C., DUNNILL, M.S., VAN MUIJEN, G.N.P. & MASON, D.Y. (1986). Human lung tumours may coexpress different classes of intermediate filaments. *J. Clin. Pathol.*, **39**, 950–954.

HSU, S.-M., RAINE, L. & FANGER, H. (1981). Use of avidin-biotin peroxidase (ABC) in immunoperoxidase techniques: a comparison between ABC and unlabelled antibody (PAP) procedures. *J. Histochem. Cytochem.*, **29**, 577–580.

LEVITT, M.L., GAZDAR, A.F., OIE, H.K., SCHULLER, H. & THACHER, S.M. (1990). Crosslinked envelope-related markers for squamous differentiation in human lung cancer cell lines. *Cancer Res.*, **50**, 120–128.

MINNA, J.D., PASS, H., GLATSTEIN, E. & IHDE, D.C. (1989). Cancer of the lung. In *Cancer: Principles and Practice of Oncology.* Devita, V.J. Jr, Hellman, S. & Rosenberg, S. (eds.), J.R. Lippincott Co: Philadelphia, pp. 591–705.

MIYAZAKI, K., MASUI, H. & SATO, G.H. (1982). Control factors for keratinisation of human bronchogenic epidermoid carcinoma cells. In *Growth of Cells in Hormonally Defined Media.* Sato, G.H., Pardee, A.B. & Sirbasku, D.A. (eds). Cold Spring Harbor Laboratory: Cold Spring Harbor, NY. pp. 657–661.

MOSS, F., BOBROW, L.G., SHEPPARD, M.N., GRIFFITHS, M., ROWE, D., BEVERLEY, P.C.L., ADDIS, B. & SOUHAMI, R.L. (1986). Expression of epithelial and neural antigens in small cell and non-small cell lung carcinoma. *J. Pathol.*, **149**, 103–111.

RICE, R.H. & GREEN, H. (1979). Presence in human epidermal cells of a soluble protein precursor of the cross-linked envelope: activation of the cross-linking by calcium ions. *Cell*, **18**, 681–694.

SAID, J.W., NASH, G., SASSOON, A.F., SHINTAKU, I.P. & BANKS-SCHLEGEL, S. (1983). Involucrin in lung tumours–a specific marker for squamous differentiation. *Lab. Invest.*, **49**, 563–568.

SALGE, U., KILIAN, P., NEUMANN, K., ELSASSER, H.-P., HAVEMANN, K. & HEIDTMANN, H.-H. (1990). Differentiation capacity of human non-small-cell lung cancer cell lines after exposure to phorbol ester. *Int. J. Cancer*, **45**, 1143–1150.

SARTORELLI, A.C. (1985). Malignant cell differentiation as a potential therapeutic approach. *Br. J. Cancer*, **52**, 293–302.